# Cellulose-Derived Nanostructures as Sustainable Biomass for Supercapacitors: A Review

**DOI:** 10.3390/polym14010169

**Published:** 2022-01-01

**Authors:** Seong Min Ji, Anuj Kumar

**Affiliations:** 1 Department of Nano Convergence Engineering, Jeonbuk National University, Jeonju 561-756, Korea; jsm10634@naver.com; 2 School of Chemical Engineering, Yeungnam University, 280 Daehak-ro, Gyeongsan 38541, Korea

**Keywords:** sustainable biomass, cellulose-derived nanostructures, nanocellulose, carbon nanofibres, specific capacitance, supercapacitors

## Abstract

Sustainable biomass has attracted a great attention in developing green renewable energy storage devices (e.g., supercapacitors) with low-cost, flexible and lightweight characteristics. Therefore, cellulose has been considered as a suitable candidate to meet the requirements of sustainable energy storage devices due to their most abundant nature, renewability, hydrophilicity, and biodegradability. Particularly, cellulose-derived nanostructures (CNS) are more promising due to their low-density, high surface area, high aspect ratio, and excellent mechanical properties. Recently, various research activities based on CNS and/or various conductive materials have been performed for supercapacitors. In addition, CNS-derived carbon nanofibers prepared by carbonization have also drawn considerable scientific interest because of their high conductivity and rational electrochemical properties. Therefore, CNS or carbonized-CNS based functional materials provide ample opportunities in structure and design engineering approaches for sustainable energy storage devices. In this review, we first provide the introduction and then discuss the fundamentals and technologies of supercapacitors and utilized materials (including cellulose). Next, the efficacy of CNS or carbonized-CNS based materials is discussed. Further, various types of CNS are described and compared. Then, the efficacy of these CNS or carbonized-CNS based materials in developing sustainable energy storage devices is highlighted. Finally, the conclusion and future perspectives are briefly conferred.

## 1. Introduction

Supercapacitors are energy storage devices that can manage higher power rates compared to batteries [1]. Supercapacitors provide the option of energy absorption along with energy transfer in seconds or tens of seconds. Supercapacitors can deliver hundreds to thousands times more power in the same volume, but cannot store the same amount of charge as batteries [2]. They can be used independently or with fuel cells or batteries where high power is required [3,4]. Due to these characteristics, they are considered a complement to batteries [5]. Supercapacitors stand to be promising candidates in numerous portable electronic devices and hybrid electric vehicles due to their long cycle of life, safe and ultra-fast charging and discharging feature, and strong thermal operating temperature.

Conductive polymers are widely used alternatives to metallic or inorganic semiconductors for fabricating pseudocapacitive materials because of their cost-effectiveness, non-toxicity, elegant electronic conductivity (10,000 S/cm), and high electronegativity. The presence of π-conjugated double bonds in conductive polymers is the backbone responsible for redox reactions with the ability to store and charge a large amount of charge [6]. The most frequently used conductive polymers in energy storage and conversion devices are polypyrrole (PPy) [6], polyaniline (PANi) [7], polyacetylene (PA) [8], poly(3,4-ethylenedioxythiophene) (PEDOT) [3], polythiophene (PTh) [3,6,7,8,9]. Conductive polymers are classified into three main types: The first is when two identical p-doped conductive polymer films are used for both electrodes, such as PANi and PANi. The second type is when two different P-doped conducting polymers are used as electrodes, such as PPy as the anode and PTh as the cathode. However, the operating potential windows of the first and second types are less than 1.0 V and 1.5 V, respectively, due to the thermodynamic water decomposition potential (1.23 V) and non-aqueous electrolytes (4.0 V). Similarly, the third type is when two different polymers, like p-doped polymers and n-doped polymers, are used for the anode and cathode, respectively. When the material is fully charged, the anode is p-doped and the cathode is n-doped; this phenomenon is highly dependent on electrochemical events. However, expansion and contraction upon cycling are major drawbacks of polymer-based electrode materials. This causes deterioration of the electrode and structural damage, thereby resulting in poor cycle stability because of the change in volume during the charging and discharging process. Fabricating composites from metal oxides or carbon-based materials can overcome these shortcomings.

To tackle the limitations of conductive polymers, various composite materials that combine electrostatic charge accumulation with fast redox reactions have been investigated. For example, carbon nanotubes embedded with PPy nanowires can retain 72% of their capacitance after 3000 cycles (1A/g) [10]. PPy reduced graphene oxide (rGO) composites can retain 85% of their capacitance after 1000 cycles [11]. However, the environmental risk of GO or carbon nanotube (CNT) synthesis and the high cost of using GO or CNT as a framework limits their applications. Biomass rich in carbon is considered a sustainable and ideal carbon source for the large-scale production of carbon due to its low cost, renewability and CO_2_ neutrality. Cellulose, the most abundant and widely available biopolymer in nature (over 50 wt% of plant biomass), could be a sustainable carbon precursor for porous carbon. Recent biomass-based carbon sources in use include lignin [12], cellulose [13], chitin [14] or chitosan [15], microorganisms [16], proteins [17], and gelatin [18]. Among them, cellulose-based composite foams, gels and aerogels have promisingly been applied to advanced energy storage devices [19,20]. However, these carbon materials typically contain mesopores and micropores, impeding the penetration and loading of conductive polymers. Therefore, the fabrication of high-performance biomass-derived carbon/conductive polymer hybrids remains a major challenge.

## 2. Supercapacitors

### 2.1. Fundamentals and Technologies

**Figure 1:**
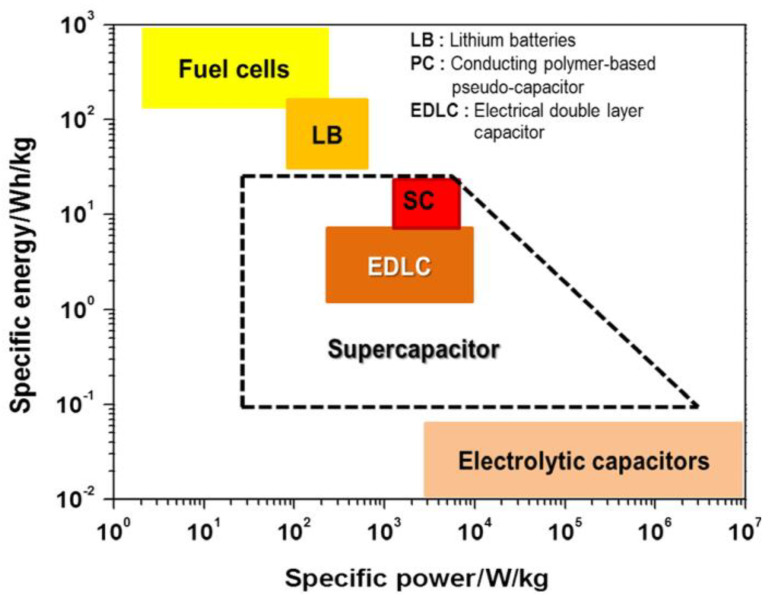
Ragone plot comparison of various energy storage technologies for energy vs. power density [21].

Figure 1 shows the energy and power densities of various energy storage and conversion systems [21]. Supercapacitors provide high power density, while fuel cells provide high energy density. Supercapacitors are distinctly classified into two types based on their charge storage mechanisms such as electric double layer capacitors (EDLCs) and pseudocapacitors. Depending on the properties of the electrode material used, supercapacitors can sometimes store energy in a capacitive manner similar to EDLCs, offering excellent energy and power densities and long-term durability [22].

**Figure 2:**
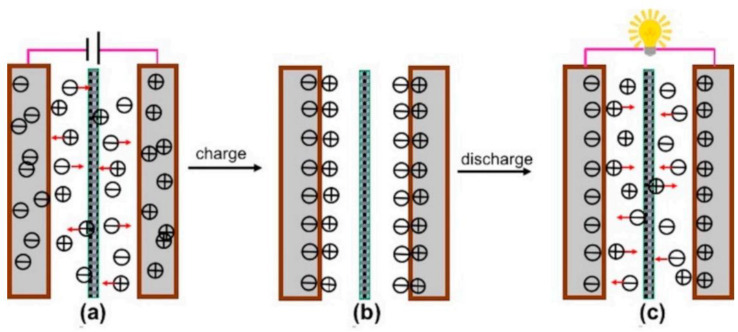
Schematic illustration of the charging process (**a**): charged state (**b**), discharging process (**c**) [26].

**Figure 3:**
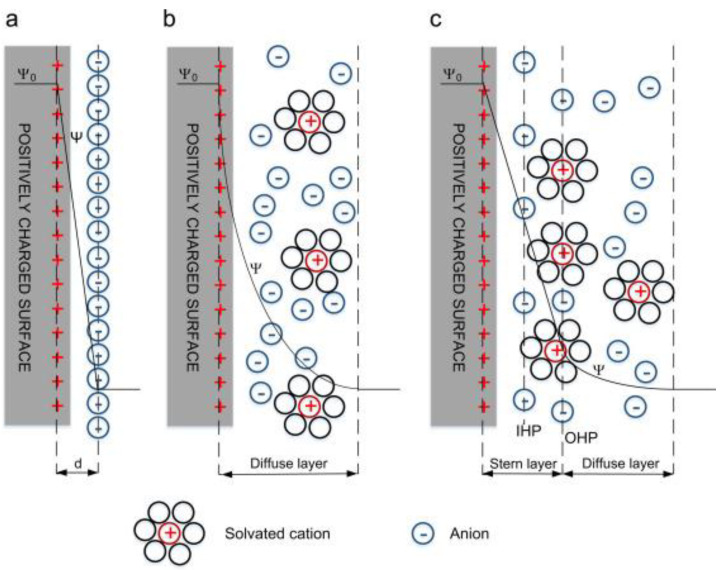
EDL model, (**a**) Helmholtz model, (**b**) Gouy–Chapman model, and (**c**) Stern model. Reprinted with permission from ref. [1]. 2016 Elsevier.

Electrical double layer capacitors (EDLCs) are a non-paradoxical process in which charge is stored through the adsorption of electrolyte ions at the electrode/electrolyte interface. In EDLC, charge accumulates at the electrode/electrolyte interface to form an EDLC structure of several angstroms (Å) balanced by counter-charges provided by the electrolytes used. Thus, EDLCs have two distinct bilayers at the anode and the cathode ends, with charging and discharging conditions as presented in Figure 2. In other words, there is no paradoxical reaction between the electroactive material and the electrolyte, so it has superior long-term durability and high power density compared to similar capacitors. The EDLC concept was first conveyed by the German physician Helmholtz in 1853, where he hypothesized that charge accumulation occurs by electrostatic attraction at the electrode–electrolyte interface [23]. Later, Gouy [24] and Chapman [25] modified Helmholtz’s EDLC model by using two layers of opposite charges at the electrode–electrolyte interface. Finally, both the Helmholtz model and the Gouy and Chapman modifications were combined together to find the ion diffusion layer, i.e., the diffusion layer as shown in Figure 3, where the dense layer is labelled as the stern layer. Thus, the total capacitance of the EDL is the combination of a small Helmholtz layer (*C_H_*) and a diffusion layer (*C_diff_*).

1Cdl=1CH+1Cdiff

**Figure 4:**
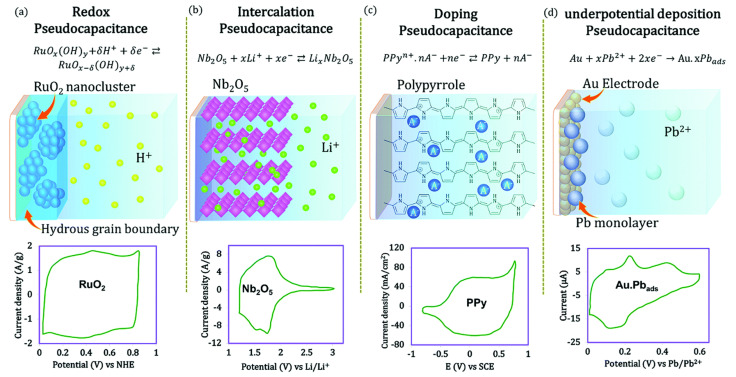
Different type of pseudocapacitances and the nature of the cyclic voltammetry (CV) curve. Reprinted with permission from ref. [22]. 2019 American Chemical Society.

In 1970s, Conway and Trasatti proposed another route for storing capacitive charge, known as pseudocapacitance. Faradic reactions are reversible and involve a change in the valence state of an electrode material as the result of a battery-like redox reaction (oxidation and reduction occurring simultaneously) [27,28]. In general, metals, metal oxides and conductive polymers are recognized as pseudocapacitive electrode materials. Pseudocapacitive materials store charge by redox reactions. Basically, batteries and supercapacitors also involve redox reactions, but the reaction kinetics are distinctly different. The charge storage mechanisms of supercapacitors fall into four groups: (1) redox pseudo-capacitance, (2) intercalated pseudo-capacitance, (3) pseudo-capacitive doping, and (4) low-potential deposition-based pseudo-capacitance, as shown in Figure 4.

In a redox supercapacitor, charge is stored through fast and reversible electron transfer across the electrode and electrolyte interface, which is expressed as

$$\mathrm{Ox}+zC^{+}+ze^{-}↔\mathrm{Red}\;C_{z}$$

where *C* represents the surface absorbed electrolyte cations and z represents the number of electrons transferred during the reaction. In intercalation pseudocapacitance, when electrolyte ions (Li^+^, H^+^, K^+^ Na^+^,…) are reversibly inserted and ejected from a tunnel or layer, they are accompanied by a paradoxical charge transfer rather than a phase change. This is rarely observed, and mostly follows a hybrid charge storage mechanism, i.e., a combination of capacitive and battery-like behaviour. The doping pseudocapacitance refers to a reversible electrochemical process in which doping and de-doping processes occur similarly to conductive polymers (polypyrrole). Underpotitiontial deposition pseudocapacitance is well known for the adsorption of hydrogen atoms into catalytic noble metals (Pt, Rh, Ru and Ir), with electrodeposition of metal cations at potentials less negative than the equilibrium potential for cation reduction. This process can be represented as:

$$M+xC^{z+}+xze^{-}↔C.M$$

where *C* is the absorbed atom, *M* is the noble metal, *x* is the number of absorbed atoms, and *z* is the valence of the absorbed voltage. For a better understanding, hydrogen potential is deposited on the noble metal (Pt). The potential must be positive in a reversible hydrogen electrode (RHE) [22,29].

### 2.2. Materials Used for Capacitors

Nanotechnology has opened a remarkable new research direction, enabling new functional materials and technologies for energy storage applications. Nanostructured electrodes present specific properties owing to their structure-dependent benefits in expediting the reaction kinetics of the diffusion activity and attaining cyclic stability. Various electrode materials with low dimensional architectures (0D, 1D, 2D, and 3D) and hierarchical and hollow nanostructures have been widely designed and applied in this area [30,31,32,33]. Electrode materials are the main components in the development of high-efficiency supercapacitors for next-generation portable electronic devices. There are four main types of electrode materials for supercapacitors: full density metal oxides/hydroxides, carbon-based materials/nanomaterials, conductive polymers (CPs), and nanocomposites of various materials. Exclusively, these materials are transition metal oxides/hydroxides such as nickel oxide or hydroxides (NiO [34] or Ni(OH)_2_ [35]), rare-earth metal oxides or hydroxides such as lanthanum oxide or hydroxides (La_2_O_3_ or La(OH)_3_) [36,37], transition metal dichalcogenides (TMDs), MXenes, carbon nanomaterials such as carbon nanotubes or graphene oxide (CNTs or GO [37]), conducting polymers, etc. These electrode materials are associated with certain advantages and disadvantages in that they are effectively used as electrode materials in supercapacitors. Therefore, the composite electrode materials made from them have shown great potential in supercapacitor applications.

Over the passage of time, metal oxides are being extensively studied as a paradoxically capacitive electrode material due to their high energy density compared to carbon-based materials and superior long-term durability compared to conductive polymers. Various metal oxides have been recognized as electrode materials for supercapacitors, such as ruthenium oxide (RuO_2_), cerium oxide (CeO_2_), manganese oxide (MnO_2_), nickel oxide (NiO), and cobalt oxide (Co_3_O_4_). Some basic requirements for metal oxides utilized as electrode materials in supercapacitors are: (1) low cost, (2) easy solubility, (3) improved electrical conductivity, (4) improved specific capacitance, (5) tendency to exist in various oxidation states.

In 1971, Trasatti and Buzzanca first discovered an alternative charge storage mechanism (pseudocapacitive) using ruthenium oxide (RuO_2_) in sulfuric acid (H_2_SO_4_) [28]. Before being investigated as a pseudocapacitive material, RuO_2_ was very popular as an oxygen-generating anode due to its electrocatalytic properties. Both forms of RuO_2_ (ie, crystalline and hydrous) exhibit excellent electrochemical behaviour. On the other hand, RuO_2_ can exhibit paradic redox kinetics in both acidic and basic media and can deliver specific capacitances of up to 1500 F/g. However, the reaction kinetics are quite different for both electrolytes. In the acidic electrolyte, rapid electron transfer is achieved because the electroadsorption of protons on the RuO_2_ surface and oxidation turned Ru(II) into Ru(IV). The reaction mechanism of acid electrolytes is expressed as:

$${\mathrm{RuO}}_{2}+{\mathrm{xH}}^{+}+{\mathrm{xe}}^{-}↔{\mathrm{RuO}}_{2-x}{\left(\mathrm{OH}\right)}_{x}$$

where the oxidation states of ruthenium can change from (II) up to (IV) (0 ≤ x ≤ 2) [22]. Ruthenium has been considered an expensive metal in the platinum group, as compared to transition metals that are inexpensively produced [38]. The high cost of this material compared to other transition metals has limited its practical applications.

Manganese (Mn) is the tenth most abundant element in the Earth’s crust in the form of ore and natural metal nodules. Mn is readily depleted in igneous and metamorphic rocks by surface water and through interactions with surface water. Mn is readily oxidized to more than 30 oxide/hydroxide minerals that are the main source of industrial Mn [39]. Manganese oxide is the most widely used transition metal oxide in the fields of catalysts, sensors, energy and wastewater treatment [40,41].

Storage behaviour of MnO_2_.

The first is the intercalation and deintercalation of electrolyte cations (C^+^: Na^+^, K^+^, Li^+^) in large amounts of MnO_2_ during the oxidation and reduction processes.

$${\mathrm{MnO}}_{2}+C^{+}+e^{-}↔\mathrm{MnOOC}$$

The second mechanism based on the electrolytic cation (C^+^: Na^+^, K^+^, Li^+^) adsorption on the MnO_2_ surface, as follows.

$${\left({\mathrm{MnO}}_{2}\right)}_{\mathrm{surface}}+C^{+}+e^{-}↔{\left(\mathrm{MnOOC}\right)}_{\mathrm{surface}}$$

Interestingly, both charge storage mechanisms followed the redox reaction from the IV to III oxidation states of manganese oxide [42].

Cerium (Ce) is one of the most studied metals in the lanthanide family [43]. Cerium oxide (ceria) has been widely investigated as a lanthanide-based electrode material for supercapacitors and catalysts due to its interesting properties such as abundance, cheapness, various forms, and high electronic conductivity [44]. Cerium oxide (CeO_2_) crystallizes favourably into CeO_2_ because it is in the most favourable oxidation state of Ce^4+^ [45]. The electron configuration is 4f15d16s2, and it cannot stay in the Ce^3+^ state by losing one 5d and two 6s electrons. The most favourable oxidation state of cerium, Ce^4+^, exists by losing one additional 4f electron [46]. A direct and rapid transformation from the Ce^3+^ to the Ce^4+^ state with the loss of negatively charged oxygen atoms is a result of the oxygen lattice, leading to structural defects (oxygen vacancies) [44,47]. Thus, oxygen vacancies cause oxygen mobility and lead to an increase in the overall electrochemical performance.

Among the various metal oxides, nickel oxide (NiO) is highly regarded as a pseudocapacitive electrode material due to its low cost, abundance, elegant thermal and chemical stability, and high theoretical specific capacitance [48]. There are two theories of redox reactions in NiO-based materials. The first is an energy storage phenomenon between NiO and NiOOH, and the other is an electrochemical reaction that occurs between Ni(OH)_2_ and NiOOH, which is expressed as:

$$\mathrm{NiO}+{\mathrm{OH}}^{-}↔\mathrm{NiOOH}+e^{-}$$

$$\mathrm{NiO}+H_{2}O↔\mathrm{NiOOH}+H^{+}+e^{-}$$

These two mechanisms suggest that the electrochemical performance of NiO-based electrodes is largely determined by the redox reaction of NiO or NiOOH in the basic electrolyte [49,50].

Spinal and cubic structures of cobalt oxide (Co_3_O_4_) are highly regarded as pseudocapacitive electrode materials [51]. Co_3_O_4_ is a black antiferromagnetic solid with a bandgap of 2.0 eV, and is mainly used as an electrode material for supercapacitors and lithium-ion batteries due to its excellent economy and corrosion resistance [52,53]. The electrochemical potential of Co_3_O_4_ is reflected in the following equations.

$${\mathrm{Co}}_{3}O_{4}+{\mathrm{OH}}^{-}+H_{2}O\;↔3\mathrm{CoOOH}+e^{-}$$

$$\mathrm{CoOOH}+{\mathrm{OH}}^{-}↔{\mathrm{CoO}}_{2}+H_{2}O+e^{-}$$

However, low cycle stability due to volume expansion and contraction during charging and discharging is a major disadvantage of Co_3_O_4_-based materials used in industrial fields [52,53]. In order to overcome these problems, various research efforts are underway, such as manufacturing various binary and ternary metal oxide composites or making composites with highly conductive base materials.

In past decades, carbon-based materials have been widely studied as electrode materials for various energy storage and conversion devices due to their ease of availability, cost effectiveness, excellent electrical and mechanical properties, high power density and corrosiveness, and resistance properties [54]. Activated carbon, graphene, and carbon nanotubes are frequently used materials for supercapacitors with electric double layer properties, unless functionalized or composited with metal oxides or sulphides [55,56].

Activated carbon is the cheapest carbon material used as an electrode material for a variety of energy storage and conversion applications. Activated carbon is made from commercially available wood, petroleum sources, and phenolic resins with surface areas of 2500–3000 m^2^/g and controlled porosity. The cycle endurance of activated carbon is very high, over 10,000 cycles, and the operating potential can be achieved at 1.0–3.0 V (both aqueous and non-aqueous electrolytes), but its relatively low specific capacitance limits its application. Furthermore, TMDs have been widely investigated for electronic devices and energy storage systems due to their higher electrical conductivity than that of oxides, distinctive electronic structure, 2D sheet-like morphology, high surface area, and multivalent oxidation states of transition metal ions [57]. TMDs are a new lineage of 2D nanosheets, typically having general formula as MX_2_ (where, M: transition metal and X: chalcogen), for example molybdenum disulphide (MoS_2_) [58] or diselenide (MoSe_2_) [59], nickel selenide (NiSe) [60], cobalt selenide (CoSe) [61]. In addition, MXenes, discovered in 2011 and known as 2D carbides and the nitrides of transition metals, have been considered a favourable electrode material for supercapacitors due to their hydrophilicity, outstanding electrochemical properties and high electrical conductivity [58,62,63]. Furthermore, the most frequently used conductive polymers in energy storage and conversion devices are PPy [6], PANi [7], PA [8], PEDOT [3], and PTh [9]. Recently, sustainable biomass-derived carbon nanostructures have shown great potential in energy storage applications. Therefore, in the further sections, we present the fundamental properties and recent advances in cellulose-derived nanostructures as a sustainable biomass for supercapacitor applications.

### 2.3. Cellulose-Based Functional Materials for Supercapacitors

**Figure 5:**
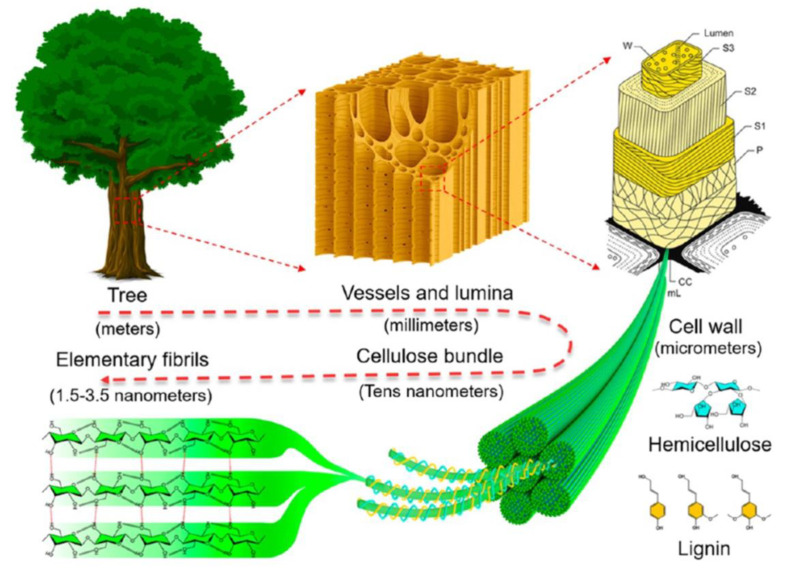
Schematic illustration of hierarchical structure of wood, from macroscopic to molecular level. Reprinted with permission from ref. [71]. 2018 American Chemical Society.

The carbon materials traditionally used in supercapacitors are mainly derived from fossil resources, so they face serious environmental problems and will be accountable for the loss of fossil resources in the near future. However, biomass rich in carbon is considered a sustainable and ideal carbon source for the large-scale production of carbon due to its low cost, renewability, and CO_2_ neutrality [64,65,66,67,68,69]. Therefore, natural biopolymers have been broadly utilized in developing flexible wearable sensors and energy devices with better performance [70]. Among them, cellulose, as the most abundant biopolymer worldwide, and which is usually extracted from agricultural wastes, plants, woods, or using bacterial strains, has been considered an attractive candidate for this purpose (Figure 5).

Cellulose, the most abundant and widely available biopolymer in nature (over 50 wt% of plant biomass) with environment-friendly and renewable characteristics, could be a sustainable carbon precursor for porous carbon. Recently, bacterial cellulose, a representative biomass material, has been widely used for the synthesis of functional carbon materials having a 3D porous structure [69,72,73]. Cellulose fibers are highly hydrophilic, flexible, renewable, biocompatible, and biodegradable biopolymers. These fibers are arranged in a 3D porous structure (i.e., fibrous-network) and have strong mechanical strength. The presence of an abundant number of hydroxyl groups on the surface of cellulose and hydrogen bonding sites make cellulose-based materials available for strong interactions with other hydrophilic substrates. Among all types of cellulosic materials, bacterial cellulose has more surface hydroxyl groups as compared to plant-based cellulose, which provides opportunities for functionalization [74]. Moreover, cellulose-based substrates exhibit light weight, ease of application, flexibility, excellent thermal and optical properties, poor heat transfer capacity (that can cause accumulation of heat and local overheating of the electronic device) and thereby insufficient stability [75].

However, these carbon materials typically contain mesopores and micropores, impeding the penetration and loading of conductive polymers. Therefore, the fabrication of high-performance biomass-derived carbon/conductive polymer hybrids remains a major challenge. In addition, while cellulose fiber is not itself conductive, it can be integrated with other conductive materials for promising application in energy devices [76]. Moreover, the efficient integration of cellulose with other substrate support for flexible supercapacitors is a tedious task which demands improvements for better outcomes. Various methods have been reported for imparting conductivity to celluloses, for example, carbonization [77], sputter deposition [78], surface coating [79], impregnation [80], electrochemical deposition [81], lamination [80], and vacuum filtration [82]. In this case, carbonization can facilitate higher conduction to cellulose but it also creates a black appearance of this carbonized substrate [76].

## 3. Cellulose-Derived Nanostructures (CNS)

Different types of CNS have commonly been prepared from cellulosic materials by using appropriate methods such as chemical, mechanical or their combinations, and specific bacteria under optimal conditions [83,84,85,86]. By using these treatments, cellulose is reduced to the nanoscale dimension in diameter or both diameter and length, which is known as nanocellulose. Nanocellulose possesses a highly crystalline structure, with linear condensed polymeric chains of α-D-glucose linked through 1–4 glycosidic bonds and it possesses excellent mechanical strength, high surface area, high aspect ratio, and, more interestingly, a large number of hydroxyl functional groups available for functionalization [84,87]. Based on the sources, extraction methods and size-dimensions, nanocelluloses can be categorized into three major terms, such as cellulose nanocrystals (CNCs), cellulose nanofibres (CNFs), and bacterial cellulose nanofibres (BCNFs). These nanostructures possess high aspect ratios, low density, low thermal-expansion, low toxicity, an inherently renewable nature, and high mechanical surfaces with ease of functionalization [88]. In some case, these nanocelluloses can further be carbonized to obtain cellulose-derived carbon nanofibres (CCBNFs). For their efficacy in terms of their usage in supercapacitors, the properties of CNS materials, including their optical and structural characteristics, are briefly presented and discussed below.

### 3.1. Cellulose Nanocrystals (CNCs)

CNCs are generally extracted from various typical sources such as plants, woods by using acid hydrolysis (e.g., sulfuric acid) [83] or enzymatic processes [89] in some cases. This acid-hydrolysis of cellulose produces highly crystalline rod-like nanostructures or nanocrystals by removing amorphous regions from the cellulose fibers. Additionally, the surface charge on CNCs is also introduced by these acid treatments and exhibits stable colloidal dispersions. Typically, CNCs have size dimensions 5–50 nm in diameter and 100–500 nm in length or longer, up to several micrometers [90,91]. In addition, CNCs possess a crystallinity percentage from 50 to 90% based on cellulosic sources and methods, and have remarkable mechanical properties (e.g., high strength: 7.5 GPa and modulus: 10–50 GPa in the transverse direction and 110–220 GPa in the axial direction) [91,92,93]. Moreover, the extensive hydrogen bonding networks in CNCs show a shear-thinning property and also can facilitate good stress-transfer effects in a polymeric matrix [93].

### 3.2. Cellulose Nanofibres (CNFs)

CNFs are prepared from different sources such as plants and wood using mechanical methods (e.g., high pressure homogenization) or in combination with chemical or enzymatic methods. In this case, the mechanical disruption of cellulose produces the defibrillation of cellulose fibers into fine fibrils, with some amorphous content depending on the methods used. Therefore, CNFs are soft and flexible fibrils that possess much lower crystallinity compared to CNCs or BCNFs. Generally, CNFs have size dimensions in the range of 10–100 nm in diameter and several micrometers in length (>10 µm). In addition, the estimated tensile modulus of CNFs is much lower than that of CNCs, approximately 30 GPa. Moreover, CNFs also show a shear-thinning property in solutions [93]. Electron storage device substrates composed of CNFs show remarkable flexibility, mechanical, and optical characteristics. The combination of high thermal conductivity fillers with CNFs provides a good approach for effective thermal management [76].

### 3.3. Bacterial Cellulose Nanofibres (BCNFs)

BCNFs are synthesized by using various bacterial strains (e.g., *Gluconacetobacter xylinus*) through the oxidative fermentation of saccharides in media (natural or synthetic) in which the bacteria are cultured under aerobic environments. The culture conditions are the critical factors in synthesizing BCNFs and they effect the morphology, crystallinity, and mechanical properties of BCNFs, which range from floccus shapes to pellicle forms [94]. Among all types of cellulosic materials, BCNFs possess maximum hydroxyl functional groups on their surface. BCNFs are composed of continuous fibers with diameters of 10–100 nm and a high crystallinity of 74–96% [93,95]. Further, the Young’s modulus of the single fibers of BCNFs is measured to be 78–114 GPa, depending on the particular technique of estimation [93,96,97]. The measured moduli values are higher than those of CNFs and are in the value range of CNCs.

### 3.4. Cellulose-Derived Carbon Nanofibres (CCBNFs)

Carbon materials are considered a potential candidate in applying electrode materials for supercapacitors due to their superior electrical conductivities, stable and adaptable structures [98]. Furthermore, carbon nanofibrous materials with high surface area and porosity and abundant oxidative groups are more effective for accessing electrolyte and electrochemical properties. Carbon nanofibres (CBNFs) can also be synthesized from biomass materials for supercapacitors [99]. In addition, compared to the synthetic carbon nanofibrous materials, the carbonization of nanocellulosic materials could be another effective approach to exploit CNS in the development of composite or hybrid electrode materials for supercapacitors [100].

## 4. Applications of CNS-Based Functional Materials in Supercapacitors

Natural polysaccharides (e.g., cellulose) and their derivatives have effectively been utilized in preparing porous carbon electrodes due to their abundance, low processing cost, and eco-friendly nature. For example, BCNFs and carboxymethylcellulose were used as carbon sources to fabricate hierarchical porous carbon (HPC) by including citric acid, exhibiting high specific capacitance (350 F/g at 15 A/g), outstanding rate capability (254 F/g at 15 A/g), and good capacitance retention (96%) after 10,000 cycles. Furthermore, a symmetric supercapacitor assembled by HPC electrodes exhibited a high specific capacitance and energy density (28 Wh/kg) in KOH aqueous electrolyte [101]. Cellulose-derived nanostructures (e.g., CNCs, CNFs, BCNFs, and CBNFs) have been promisingly utilized to develop electrodes for supercapacitors. The efficacy of CNS-based research studies is described and discussed comparatively in the next sections. For example, a supercapacitor made of renewable cellulosic papers gives a new insight on paper electronics by including the electroadsorption of paper, particularly CNFs. These amorphous dry amorphous CNF (ACNFs) capacitors are entirely different from conventional wet electric double-layer capacitors (EDLCs) and lithium-ion batteries (LIBs) that are regulated through ion diffusion. Therefore, Fukuhara et al. investigated the effect of electroadsorption on an ACNF supercapacitor and showed a high amount of stored electricity (221 mJ/m^2^, 13.1 W/kg). Also, ACNFs captured both positive and negative electricity from the vacuum and atmosphere, and could illuminate a red LED for 1 s after charging (2 mA at 10 V) [102].

### 4.1. CNS/Metallic Oxide or Hydroxide-Based Supercapacitors

Transition metal oxides have shown great potential as electrode materials for flexible supercapacitors, owing to their high specific capacitance and surface area-to-volume ratio, with cobalt oxide (Co_3_O_4_) NPs in particular having been explored in the field of CNFs. However, the aggregation and low intrinsic conductivity of Co_3_O_4_ NPs limits their electrochemical uses for commercialization. To prevent these disadvantages, a cost-effective sol-gel approach has been utilized to produce Co_3_O_4_ NPs instead of low-cost and eco-friendly 1D hydrophilic CNFs. The as-produced system exhibited high conductivity (high specific capacitance: ~214 F/g at 1.0 A/g with capacitance retention of ~94% even after 5000 cycles) compared to that of neat CNFs and Co_3_O_4_ electrodes in aqueous electrolyte. In addition, these binder-free 1D Co_3_O_4_@CNFs as flexible paper-like electrodes (carbonized at 200 °C for 20 min under H_2_/Ar ambience) exhibited high specific capacitance (80 F/g at 1.0 A/g) with better energy density (10 Wh/kg) in the gel electrolyte [103]. Moreover, CNFs and the hybrid zeolite imidazole framework (HZ) have become promising functional materials, providing adaptable physicochemical properties in combination. Therefore, CNFs and Co-containing zeolite framework-based composite were fabricated by using in situ and environment-friendly chemical processes, followed by pyrolysis. This composite was formed with Co NPs decorated on largely graphitized N-doped nanoporous carbon (NPC) enclosed with carbon nanotubes (CNTs) manufactured by the direct carbonization of HZ. This CNFs-HZNPC composite (electrodes) exhibited better electrochemical properties (specific capacitance: 146 F/g at 1.0 A/g with a capacitance retention of around 90% over the 2000 cycles at 10 A/g) suitable for supercapacitor uses [104]. These studies demonstrate the effectiveness of this transition for the development of cellulose based supercapacitors.

Manganese dioxide (MnO_2_) is marked as an active functional material for supercapacitors due to its high specific capacitance (theoretical), natural abundance, low cost, and non-toxicity [105]. However, its poor electrical conductivity prevents its thorough utilization during charge/discharge cycles. Therefore, the direct growth of MnO_2_ onto electrically conductive NPs or substrates is a promising design approach for better applications [106,107]. Although the growth of MnO_2_ on graphite papers through chemical reactions is very limited, CNF-coated graphite papers interestingly improved the thickness of grown MnO_2_ layers, and thereby volumetric-specific capacitance. Moreover, a symmetric supercapacitor showed a highest volumetric energy density of 10.6 mWh/cm^3^ at 0.11 W/cm^3^ power density, which is better than that of previous MnO_2_-based symmetric or even asymmetric devices [108]. Further, the oxidation of nanocelluloses (e.g., CNFs) might have a promising effect on supercapacitor properties. For example, 2,2,6,6-tetramethylpiperidine-1-oxylradical (TEMPO)-oxidized CNFs were pyrolyzed and then 3D-composite aerogels of TEMPO-CNFs and manganese dioxide (MnO2) were fabricated through a simple hydrothermal process. TEMPO-CNFs/MnO_2_ with the content of 20.8% MnO_2_ showed most remarkable electrochemical properties (specific capacitance: 171.1 F/g at 0.5 A/g with capacitance retention of 98.4% after 5000 cycles at 3 A/g). Furthermore, an asymmetric supercapacitor assembled by using TEMPO-CNFs/MnO2 (as the positive electrode) and activated carbon (as the negative electrode) exhibited an energy density of 8.6 Wh/kg (at a power density of 619.2 W/kg) and could still last 4.13 Wh/kg at 6.8 kW/kg power density. Moreover, an excellent capacitance retention of 99.4% of the first cycle after 4500 cycles (at 3 A/g) was observed [109].

### 4.2. CNS/Conductive Carbon Materials-Based Supercapacitors

**Figure 6:**
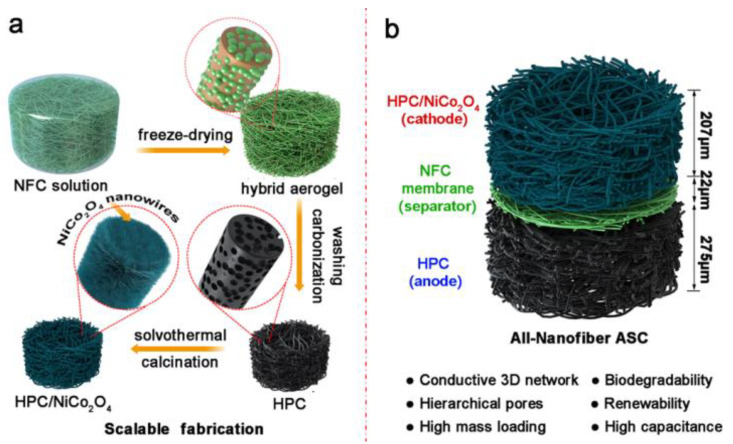
Schematic representation of the (**a**) fabrication of nanocellulose-derived HPC and HPC/NiCo_2_O_4_ composite aerogels and (**b**) their asymmetric assembly into an all-nanofibre ASC device. Reprinted with permission from ref. [112]. 2019 American Chemical Society.

Conductive carbon materials, especially nanomaterials, are considered very effective in energy storage applications and possess remarkable properties, but their high production costs may limit extensive utilization. Therefore, low-cost activated carbons (ACs) have been used to improve the characteristic performance without considerable increases in cost. In this case, a free-standing CBNFs/AC film after the carbonization of CNFs (as low-cost renewable material) is an effective design approach, with a high AC-CNFs affinity that is much stronger than the conventional physically mixed AC/nanocarbon composite and which remarkably decreases the contact resistance in the composite system. The network of carbonized CNFs provides significantly superior electron transport efficiency than AC particles [110]. Similarly, graphene fibre-based electrodes are also considered promising candidates for supercapacitors in energy storage applications, but their low electrochemical properties due to the re-stacking of graphene nanosheets and hydrophobicity to electrolytes are some disadvantages. Here, Chen et al. presented a strategy to fabricate hybrid fibres from graphene oxide (GO) and CNCs through non-liquid-crystal spinning followed by the4 chemical reduction. The hybrid GO/CNCs (100/20) fibres exhibited improved hydrophilicity (contact angle of 63.3°), mechanical properties (strength: 199.8 MPa), capacitive performance (208.2 F/cm^3^), and conductivity of 64.7 S/cm. Furthermore, a supercapacitor assembled from this composite fibre showed a high energy density (5.1 mWh/cm^3^) and power density (496.4 mW/cm^3^), remarkable flexibility and bending stability [111]. In addition to this study, a high performance all-nanofibre asymmetric supercapacitor (ASC) was fabricated by the assembly of a nanocellulose-derived hierarchical porous carbon (HPC) as the anode, a mesoporous nanocellulose separator, and a HPC/NiCo_2_O_4_ as the cathode, with nanocellulose carbon as supporting matrix (Figure 6). This all-nanofibre ASC exhibited high electrochemical properties (64.83 F/g at 0.25 A/g and 32.78 F/g at 4.0 A/g) [112].

**Figure 7:**
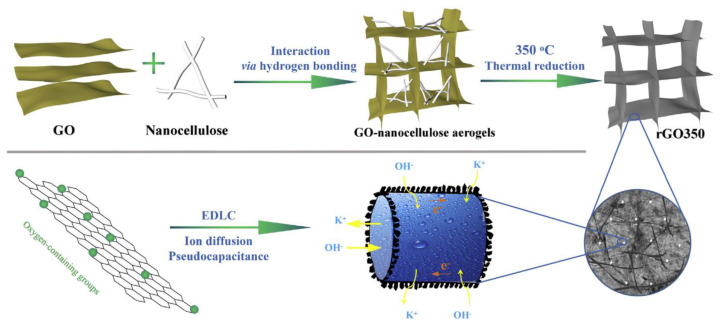
Schematic representation of the synthesis and ionic and electronic transport research mechanisms of rGO aerogel as a capacitor electrode within a KOH electrolyte. Reprinted with permission from ref. [115]. 2017 Elsevier.

The interweaving of biocompatible nanofibres with conductive carbon nanomaterials is an effective design approach to producing porous and stretchable binder-free flexible electrodes with high capacitive performance. On considering this issue, in a study, carbon nanotubes (CNTs) were used and anchored with redox juglone (J) onto their surface for interweaving with BCNFs as stretchable non-woven porous matrix. This self-standing J11-CNTs-BCNFs (11:1:5) composite exhibited the highest specific capacitance (461.8 F/g at 0.5 A/g): over 5 times higher than that of CNTs-BCNFs and a specific capacitance retention of 87.1% after 10,000 cycles at 10 A/g. Further, all solid-state ASCs assembled with AC as the negative electrode showed remarkable tolerance to bending (both time and angle) and high specific capacitance retention of 82.4% after 10,000 cycles at 10 A/g. Moreover, the ASC exhibited a maximum energy density of 41.9 Wh/kg at a power density of 1.0 kW/kg, which is better than that of most CNT-based devices [113]. In another study, the composites of iron oxide (Fe_2_O_3_)@N-multiwalled CNTs and Fe_2_O_3_)@N-multiwalled CNTs/CNCs were fabricated by a hydrothermal reduction process. These composite electrodes exhibited a maximum specific capacitance (162 F/g and 562 F/g) at the current density of 0.5 A/g, and a capacitance retention of 94.6% after 5000 cycles (cyclic stability) [114]. Furthermore, in a study, reduced-GO (rGO aerogels were synthesized by using a nanocellulose (gelator)-assisted low temperature (350 °C) thermal treatment at a low concentration dispersion (2.85 mg/mL) of rGO (Figure 7). The as-obtained compressed rGO aerogels (with amorphous CBNFs in rGO sheets) exhibited a high discharge capacitance of 270 F/g at 1.0 A/g without any binder or conductive additive. Moreover, the pseudocapacitance of the rGO electrode significantly accounted for 40.7% of its overall capacitance [115].

Similarly, reduced-GO (rGO) nanosheets decorated with tin oxide (SnO_2_) were dispersed into CNF aerogels as a framework for a supercapacitor through a facile hydrothermal reduction process followed by a freeze-drying method. This flexible CNF/rGO/SnO_2_ electrode film exhibited high specific capacitance (4.314 F/cm^2^ at 1 mA/cm^2^) in 1 M H_2_SO_4_ and excellent capacitance retention of 60.47% at 10 mA/cm^2^ after 2000 cycles [116]. In another study, rGO nanosheets were utilized with CNFs and fabricated rGO/CNF composite films through a combination of filtration and chemical reduction. Here, rGO improves conductivity and the CNFs provide flexibility, improved mechanical properties, and prevent the stacking of the rGO nanosheets. Therefore, the composite film, assembled into an asymmetric supercapacitor, showed remarkable cyclic stability, rate performance, good mechanical strength and flexibility, high specific capacitance (120 mF/cm^2^), energy density of 5361 Wh/cm^2^, and power density of 193 mWcm^2^ [117]. Similarly, Chen et al. fabricated a composite aerogel made of biomass carbon/rGO/CNFs through a one-step self-assembly process, where negatively charged CNFs acted as the binder between the biomass carbon and the rGO nanosheets. This composite aerogel exhibited high specific surface area (1007.9 m^2^/g), high conductivity, outstanding mechanical strength of 240 kPa, and an efficient MnO_2_ deposition ability (33.9 mg/cm^2^). Moreover, the use of this composite aerogel in fabricating a self-supporting supercapacitor showed excellent capacitive property (4.8 F/cm^2^) [118]. CNFs themselves have low utilization efficiency and poor conductivity. Therefore, in a study, non-carbonized CNFs/graphene quantum dot (GQD)-composite film with good flexibility sensitive to human body movement was developed through electrolysis and liquid dispersion. The as-developed composite film exhibited remarkable electrochemical storage properties (specific capacitance: 118 mF/cm^2^ even at an ultrahigh scan rate (1000 mV/s) and high capacitance retention: >93% at various current densities after 5000 cycles). In addition, the assembled supercapacitor showed high power (782 mW/cm^2^) and energy density (5961 Wh/cm^2^) at the same time [119]. These studies highlight the importance of graphene oxide and reduced graphene oxide in enhancing the conductivity of CNSs that gives a cushion in developing CNS based supercapacitors.

### 4.3. CNS/Conductive Polymers-Based Supercapacitors

**Figure 8:**
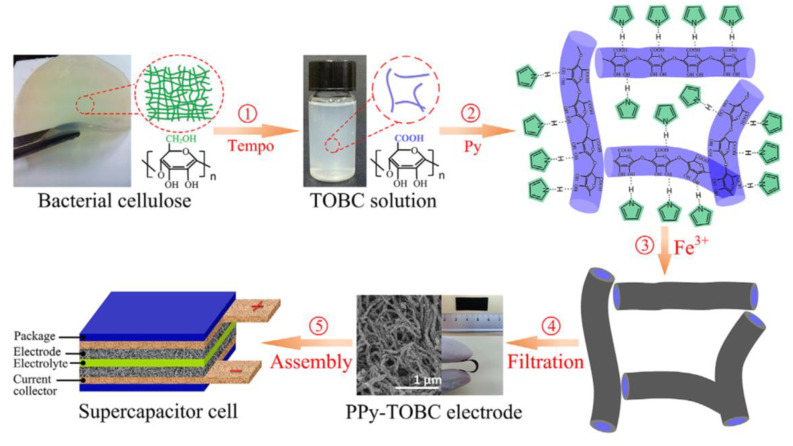
Schematic process of the fabrication and layered structure of supercapacitor cell. Reprinted with permission from ref. [124]. 2016 Elsevier.

In recent years, cellulose and conducting polymer (such as polyaniline, polypyrrole, polythiophene) based composites with nanostructures have seen promising applications in batteries and energy storage devices. Various conducting polymers have been utilized for this purpose. Among them, polyaniline (PANI) has been used extensively due to its easy synthesis, good environmental stability, facile doping/de-doping chemistry, reasonable and controllable electrical conductivity, affordability and redox properties [120]. On considering this polymer, Wang et al. 2012 developed BCNFs/PANI nanocomposite through the in situ polymerization of aniline onto BCNF nanofibrous membranes. The as-obtained nanocomposite showed an ordered, flake-like nanostructure morphology and achieved remarkable electrical conductivity (upto 5.1 S/cm) with a mass-specific capacitance of 273 F/g at a current density of 2.0 A/g [121]. In another study, CNFs were used to tailor the morphology and doping of grown PANI worm-like nanorods during in situ polymerization. In this case, a maximum specific capacitance of 421.5 F/g at 1.0 A/g was attained for the CNFs (20%)/PANI composite electrode. Also, a good capability rate and energy/power density balance were observed by using the composite electrodes. Moreover, all-solid-state supercapacitors assembled from CNF/PANI composite electrodes exhibited remarkable electrochemical properties and capacitance retention after 1000 cycles due to their mechanical flexibility [122]. Further, N-functionalized CBNFs were obtained by carbonizing polypyrrole (PPy)-coated CNFs, which were prepared by electrospinning, deacetylation of electrospun cellulose acetate NFs, and PPy polymerization. A supercapacitor electrode fabricated from N-CBNFs and a mixture of N-CBNFs and Ni(OH)_2_ exhibited high specific capacitance values of ~236.0 F/g and ~1045.0 F/g, respectively. Furthermore, a fabricated asymmetric supercapacitor was developed, comprising N-CBNFs and N-CBNFs/Ni(OH)_2_ as the negative and positive electrodes, respectively. The supercapacitor showed an energy density of ~51.0 Wh/kg, maximum power density of ~117.0 kW/kg, and excellent capacitance retention of ~84% after 5000 cycles [123]. Similarly, a Ppy-coated core-shell TEMPO-BCNFs composite network-based flexible supercapacitor was developed through the in situ oxidative polymerization of pyrrole with iron (III) chloride on TEMPO-BCNFs in an aqueous medium (Figure 8). The as-developed electrode showed high porosity (101 m^2^/g) and conductivity (~6.63 S/cm). In addition, a Ppy-TEMPO-BCNFs supercapacitor cell as prepared with polyvinylidene fluoride (PVDF)-EMIMBF4 (1-Ethyl-3-methylimidazollium tetrafluoroborate) polymeric electrolyte, exhibited specific capacitance of 153 F/g and an energy density of 21.22 Wh/kg at 0.2 A/g. This supercapacitor showed remarkably good capacitance retention (~93%) after 100 cycles, as well as good bending stability [124].

Poly(3,4-ethylenedixythiophene) (PEDOT) has also appeared as a favorable pseudocapacitor electrode material for use in supercapacitors [125] over other conductive polymers, due to its better environmental stability and high electrical conductivity [126]. However, charge and discharge stability are poor in polymer-based pseudocapacitors during the doping and de-doping process [127]. Therefore, Ravit et al. prepared PEDOT/CNCs super capacitor electrode films through an electrochemical polymerization method. The obtained PEDOT/CNC film electrode exhibited interconnected network-like surface morphology, the highest specific capacitance (117.02 F/g), an energy density of 11.44 Wh/kg, and a power density of 99.85 W/kg, respectively at 0.2 A/g (current density), with a capacitance retention of 86% after 1000 cycles [128]. Conductive polymers could further be explored in the development of supercapacitors. The association of conducting polymers with conductance enhancers like CNT or rGO could further be explored for high conducting supercapacitors.

### 4.4. CNS/Heteroatom Dopant-Based Supercapacitors

Various new design approaches have been applied in fabricating heteroatom-doped nanostructured materials by replacing some atoms with heteroatoms (e.g., boron (B), nitrogen (N), phosphorous (P), or sulfur (S)) to modify their electron–donor characteristics and thereby their surface chemical and electrical properties. On consideration, 3D P-doped, N/P co-doped, and B/P co-doped CBNFs networks were fabricated by the pyrolysis of BCNFs submerged in aqueous solutions of H_3_PO_4_, NH_4_H_2_PO_4_, and H_3_BO_3_/H_3_PO_4_, respectively. Among them, N/P co-doped CBNFs showed good supercapacitive properties [129]. In another study, the doping of N (3.9 atom%), P (1.22 atom%), S (0.6 atom%), and N/P/S co-doped pyrolyzed BCNFs (PBCNFs) exhibited specific capacitance of 255 F/g at 1.0 A/g and energy density of 8.48 Wh/kg at 1.0 A/g, with 489.45 W/kg power density. This performance was better that that of N/P-PBCNFs and N/S-PBCNF super capacitors [130].

Liu et al. developed an N/P-doped self-supporting carbon electrode through a combined templating/activating co-assisted carbonization process and vacuum filtration. In this study, a glucose precursor was first transformed into N/P-doped carbon nanosheets (NPCNs) with high specific surface area of 2073 m^2^/g and rich heteroatom-doping with P (2.1 atom% using phosphorous oxide) and N (4.1 atom% using dicyandiamide). Then, a dense free-standing electrode (NPCNs-f) was manufactured through vacuum filtration using NPCN and conductive CNFs. This free-standing electrode showed a high capacitance (318 F/g at 1.0 A/g) and maintained 188 F/g at 100 A/g in an alkaline electrolyte. Furthermore, an asymmetric supercapacitor composed of NPCN-f (as the negative electrode) and NPCN/MnO2-f syntheiszed by a self-regulated redox method as the positive electrode exhibited a high energy density (41.5 Wh/kg at 182.0 W/kg power density) and remarkable capacitance retention of 93% after 10,000 cycles in a neutral electrolyte [131]. Surface area plays an important role in developing conductive CNFs, and with the application of doping materials, their conductivity could be remarkable improved. Further studies based on doping different materials could further improve the conductance of CNFs.

### 4.5. CNS/TMDs-Based Supercapacitors

Portable and flexible electronic devices are proliferating today. Therefore, it is very imperative to develop a low-cost, light-weight, sustainable, and flexible supercapacitor with superior electrochemical properties and high operational safety. In this case, MoS_2_, as a family member of TMDs and CNFs, has attracted attention in supercapacitor applications, and it has been reported that a 2D/0D electrode composed of MoS_2_/tin sulphide (SnS_2_) quantum dots on flexible cellulose paper exhibited good performance as a high-performance flexible supercapacitor for wearable electronic applications [132]. In this way, Lv et al. developed an electrode material and charge collector composed of a CNF/MoS_2_/reduced graphene (rGO)-based hybrid aerogel film by using supercritical CO_2_ drying for a new type of all-solid-state flexible supercapacitor with sulfuric acid (H_2_SO_4_)/poly(vinyl alcohol) (PVA) gel as the electrolyte and separator. The as-developed electrode exhibited outstanding electrochemical properties, such as specific capacitance of 916.42 F/g, capacity retention of 98% after 5000 cycles at a current density of 0.5 mA/cm^2^. In addition, areal capacitance of 458.2 mF/cm^2^, areal power density of 8.56 mW/cm^2^ (4.3 kW/kg), and energy density of 45.7 mWh/cm^2^ (22.8 Wh/kg) were measured from the supercapacitors [133]. In another study, this research group carbonized these CNFs to cCNFs (as carbon nanosphere fibres: CNPFs) and developed freestanding and highly porous aerogel films of cCNFs/MoS_2_/rGO as electrodes and H_2_SO_4_/PVA as electrolytes. The as-obtained all-solid-state flexible supercapacitors showed remarkable bending ability with high specific capacitance of 1144.3 F/g at 2 mV/s and good cyclic stability (capacitance retention of 98%) after 10,000 cycles at a current density of 5 mA/cm^2^. Additionally, high energy density of 57.5 µWh/cm^2^ (28.8 Wh/kg) and power density of 29.1 mW/cm^2^ (14.5 kW/kg) were obtained [134].

### 4.6. CNS/MXene-Based Supercapacitors

Ti_3_C_2_T_x_, as the first member of the MXene family reported in 2011, has become a promising material. The re-stacking of Ti_3_C_2_T_x_ flakes unavoidably sacrifices the electroactive sites, and therefore the properties of Ti_3_C_2_T_x are_ significantly diminished with the increasing thickness of the electrode [135,136]. In one study, a flexible MXene (Ti_3_C_2_T_x_)/CNFs/porous carbon (PC) composite 3D film with a porosity of 574.5 m^2^/g and conductivity of 83.1 S/cm was fabricated through a simple vacuum filtration process. This composite 3D film possessed ample micro pores for charge storage and a substantial amount of meso-/macropores for rapid ion diffusion, while 2D Ti_3_C_2_T_x_ provided remarkably high conductivity (2.6 × 10^3^ S/cm) and a good film-forming capability, and 1D CNFs ensured high mechanical performance (tensile strength: 38.6 MPa). Here, CNFs and PC enhanced the interlayer distance between the Ti_3_C_2_T_x_ flakes, which led to rapid ion transportation. Further, this free-standing film composite was utilized to prepare a quasi-solid-state supercapacitor (ultra-thickness: 0.2 mm) with high flexibility, high areal capacitance (143 mF/cm^2^ at 1.0 mA/cm^2^), high energy density (2.4 µWh/cm^2^ at 17.5 µW/cm^2^), and high capacitance retention (~50%) even after enhancing the power density by 100-times [137]. In another study, delaminated Ti_3_C_2_T_x_ flakes were modified by alkalization and post-annealing to remarkable fluorine (-F) and hydroxyl (-OH) functional groups. Therefore, inspired by nacres, modified Ti_3_C_2_T_x_ flakes were combined with soybean stalk-derived CNFs that enhanced its mechanical performance (tensile strength: 53.9 MPa), prevented the dense packing of Ti_3_C_2_T_x_ flakes, and facilitated the transport of electrolyte ions. The optimized composite film showed high electrical conductivity of 24,930 S/m and better electrochemical properties for supercapacitors and zinc-ion capacitors. Further, this film, under a quasi-solid-state supercapacitor design, furnished high capacitance (303.1 and 211.4 F/g at 1.0 and 10.0 mA/cm^2^), remarkable capacitance retention of 92.84% over 10,000 cycles, and considerable reasonability to bending deformations [138].

### 4.7. CNS/Multicomponent Materials-Based Supercapacitors

Carbon nanofibres (CBNFs) have several advantages that make them very appealing and functional materials in fields like energy storage, due to their multifunctionality, remarkable mechanical properties, and electrical and thermal characteristics. However, biomass-derived CBNFs provide an economically efficient alternative to expensive, petroleum-derived CBNFs. Therefore, sustainable biomass has shown tremendous potential in energy storage applications, such as supercapacitors. In one study, a skin secretion of Andrias davidianus (SSAD) as a bio-nitrogen source was used to dope carbon aerogels obtained from CNCs that were very effective in the dispersion of SSAD in water. In this study, honeycomb-structured nanofibrous carbon aerogels were produced through unidirectional freeze-drying of a mixture of SSAD/CNCs/CNFs, followed by a high-temperature carbonization process (up to 800 °C). The resulted carbon aerogels showed remarkable elasticity under repeated compression and release cycles. Even, after 500 compression and release cycles, the obtained supercapacitor can still possess high capacitive properties, indicating its advantages in longevity and electrochemical stability [139]. In another study, 3D interconnected hierarchical porous carbon aerogels (CBNFAs) were fabricated through the pyrolysis processing of CNFs. The CBNFAs-17% electrode showed an ultrahigh capacitance of 440.29 F/g at 1.0 A/g, remarkably better than that of the most reported biomass-derived carbon materials. In addition, it possessed a significant rate ability of 63.29% at 10 mA/cm^2^, high areal energy density of 0.081 mWh/cm^2^, and outstanding capacitance retention of about 100% after 7000 cycles [140]. In another study, BCNFs-derived CBNFs were modified by polydopamine (PDA), which deteriorated the surface area and the pore volume but improved the wettability of the surface. Then, ferrous ion (Fe^2+^) was incorporated as a redox additive that significantly amplified the capacitive performance. The optimized CBNFs/PDA-Fe^2+^ composite system exhibited high capacitance (219 F/g) at 10.0 A/g and high energy density (10.07 Wh/kg) at 1 kW/kg power density. Moreover, it provided favorable long-term capacitance retention of up to 95%. PDA and Fe^2+^ collectively enhanced its electrochemical properties [141]. These studies highlight the features of nanocellulose-derived carbon, which includes low environmental effects, sustainability, and high specific surface area. Furthermore, Wang et al. prepared freestanding N-doped CBNF membranes obtained from cellulose acetate (CA) and soy protein isolate (SPI) through electrospinning, regeneration, and carbonization processes. In this study, the influence of carbonization temperature (700, 800, and 1000 °C) on the CBNFs’ microstructure and electrochemical characteristics was demonstrated. The obtained CA/SPI-derived heteroatom-doped CBNFs (800 °C) exhibited high specific capacitance (219.3 F/g at 0.2 A/g in 6 M KOH) and good cycling stability (98.9% capacitance retention after 50,000 cycles at 20 A/g). Moreover, a single supercapacitor assembled in series was able to light up red light-emitting diodes (LED) for 45 s [142].

The combined use of cellulose-derived nanostructures, conducting materials or polymers, with or without metallic aspects, facilitates a synergistic effect on the physicochemical and electrical properties of the developed supercapacitor electrodes. For example, a flexible and free-standing supercapacitor film electrode composed of BCNFs, PANI, and graphene was fabricated using a simple chemical polymerization and filtration method. This composite system exhibited substantial areal capacitance (4.16 F/cm^2^), high flexibility, and outstanding tensile strength (65.4 MPa). In addition, an symmetric supercapacitor coupled with a BCNF/graphene/PANI film showed good bending stability, high areal capacitance of 1.32 F/cm^2^, and energy density of 0.12 mWh/cm^2^ [143]. In another study, a BCNFs/graphene/PANI composite electrode was fabricated and used to produce a foldable all-solid-state (ASS) supercapacitor. The as-obtained composite exhibited high areal capacitance (3.65 F/cm^2^ at 5.0 mA/cm^2^) and bending stability. The assembled ASS supercapacitor provided outstanding capacitance (1389 mF/cm^2^) and energy density (9.80 mWh/cm^3^) at 2 mA/cm^2^ and 89.8% capacitance retention after 5000 cycles [144].

Additionally, Ppy as a conducting polymer, and reduced-GO (rGO) conductive nanosheets were used with CNFs and the films were fabricated through the vacuum filtration and chemical reduction methods. In this case, the developed composite film exhibited a sandwich-like construction and the bulk Ppy was enveloped in an rGO/CNFs framework that resulted in a free-standing and highly flexible supercapacitor electrode. The supercapacitor showed a specific capacitance of 304 F/g and 81.8% capacitance retention after 1000 cycles, which was higher than that of individual rGO or Ppy films, or a randomly mixed film with identical components. Moreover, a solid-state symmetric supercapacitor was assembled using two slabs of Ppy@rGO/CNFs films as electrode and 1 M H_2_SO_4_-saturated CNFs membrane as electrolyte separator. This sandwich-like system showed high specific capacitance (625.6 F/g at 0.22 A/g), 75.4% capacitance retention after 5000 cycles, and high energy density of 21.7 Wh/kg at power density of 0.11 kW/kg [145]. Furthermore, TEMPO-CNFs were used with Ppy and rGO through the same procedure described above to prepare core-shell structured Ppy@TEMPO-CNFs/rGO microfibers for all-solid-state (ASS) wearable supercapacitors. These as-developed microfibers exhibited remarkable mechanical properties (559 MPa) and were assembled into symmetrical ASS fibre-shaped supercapacitors that provided excellent electrochemical performance (647 mF/cm^2^ at 0.1 mA/cm^2^), capacitance retention of 92.5%, coulomb efficiency of 92.6% after 10,000 cycles, and outstanding flexibility (no remarkable deterioration in capacitance after 5000 bending cycles) [146]. Inspired by the Chinese pigskin jelly cuisine, Wu et al. utilized directly it as conductive hydrogel matrix and developed a flexible conductive hydrogel by adding TEMPO-nanocellulose (NC) stabilized CNTs into a pigskin matrix (PS). The developed TEMPO-NC@CNTs/PS conductive hydrogel was applied as a flexible, conductive sensor to detect human body movements. This conductive hydrogel could swiftly and precisely respond to cyclic tensile/pressure forces with stable and repeatable resistance change signals, as well as perform real-time detection of the movements of different human body parts. Furthermore, specific capacitance of 65 F/g and capacitance retention of 60% after 2000 cycles were measured [147].

**Figure 9:**
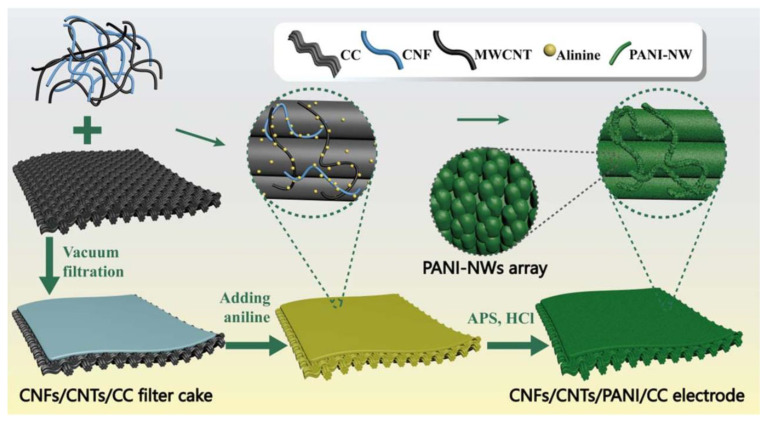
Schematic of the preparation process of CNFs/CNTs/PANI-NW/CC electrodes. Reprinted with permission from ref. [152]. 2018 IOP Publications.

Similarly, TEMPO-BCNFs were utilized with Ppy and rGO to fabricate flexible fibre-based supercapacitors composed of hierarchical Ppy@TEMPO-BCNFs/rGO microfibers through wet spinning and in situ polymerization. This composite electrode system exhibited synergistic effects with outstanding specific capacitance of 391 F/g at 0.5 A/g, and this fibre-based supercapacitor demonstrated capacitance of 259 F/g at 0.2 A/g. Moreover, the fibre-based supercapacitor showed a high energy density of 8.8. mWh/cm^3^ at a power density of 49.2 mW/cm^3^ and a high power density (429.3 mW/cm^3^) at an energy density of 4.1 mWh/cm^3^, which are superior properties to those of most reported graphene fibre-based supercapacitors [148]. In another study, PPy and CNTs were utilized with BCNFs to fabricate a hierarchical core-sheath electrode through a self-assembly process. In this case, BCNFs prevented the aggregation of CNTs, remarkably improving the wettability of the supercapacitor, and facilitating the diffusion of electrolyte ions and thereby their electrochemical properties. An assembled all-solid-state supercapacitor showed a high energy density of 8.3 Wh/kg at power density of 47.3 W/kg, a power density of 454.5 W/kg at an energy density of 4.2 Wh/kg, and outstanding cyclic retention and bending stability [149]. Similarly, a layer assembly of PPy and bimetallic hydroxide (NiMn-layered double hydroxide) was applied onto a BCNF matrix through in situ layer-by-layer dispositions, and the optimized NiMn-LDH/Ppy@BCNFs electrode material achieved high specific capacitance of 653.1 C/g at 1.0 A/g. In addition, hybrid supercapacitor produced by the assembly of NiMn-LDH/Ppy@BCNFs as the cathode and Fe_3_O_4_@*carboxylated*-MWCNTs as the anode exhibited stable properties with a high energy density (29.8 Wh/kg) at the power density of 299.0 W/kg [150]. Flexible and self-healable electro-conductive hydrogels are also an important type of soft electrodes for supercapacitor applications. For this purpose, the use of nanohybrids of CNTs and CNFs with a poly (vinyl alcohol)-borax system (PVAB) to fabricate free-standing CNTs/CNFs-PVAB composite hydrogels was considered. This CNTs/CNFs-PVAB composite hydrogel exhibited a compression stress (~93 kPa), and storage modulus (~7.12 kPa) that were 2.7 and 1.9-times higher than that of CNF/PVAB. This composite system also showed low density (~1.1 g/cm^3^) and high water amount (~95%), pH-sensitivity and intrinsic moldability, and 20 s self-healing ability. Moreover, a solid-state supercapacitor assembled by CNTs/CNFs-PVAB hydrogel provided specific capacitance of 117.1 F/g and capacitance of 96.4% after 1000 cycles. This self-healable and flexible supercapacitor exhibited outstanding capacitance retention of ~98% after 10 breaking/self-healing cycles and capacitance retention of ~95% after 1000 cycles under various deformations (twisting, bending and folding) [151]. Furthermore, Zhang et al. utilized four components: CNFs, MWCNTs, PANI, and carbon cloth (CC), to fabricate a 3D porous film-current collector as a supercapacitor electrode through vacuum filtration followed by the in situ polymerization of PANI nanowires (PANI-NW) on the surface of the CNFs, CNTs, and CC. Here, CC was utilized as a flexible current collector in the electrode (Figure 9). The results showed a low charge transfer resistance (1.50 Ω), outstanding specific capacitance (318 F/g) at 10 mA/s, and capacitance retention of 72.09% after 1000 cycles [152]. The development of nanocellulose-based supercapacitors requires the intense research and development of innovative conducting polymers to aid in the upgradation of supercapacitors.

**Figure 10:**
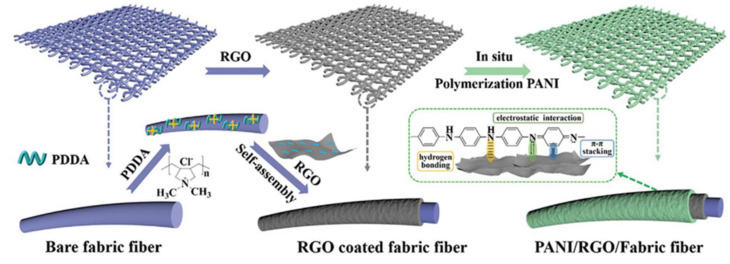
Schematic representation of a PANI@rGO/PMFT. Reprinted with permission from ref. [155]. 2021 John Wiley and Sons.

An ASS supercapacitor is generally composed of two flexible electrodes with a hydrogel electrolyte in between has remarkable features of fast charging/discharging, high power density, long cycle-life, and extensive deformation [153]. The flexible electrode is the key factor in ASS supercapacitors. In these cases, natural/artificial fibre textiles are considered highly effective due to their remarkable physicochemical characteristics, wearable properties, and 3D porous textile network [154]. The major challenge in fabricating flexible ASS supercapacitors is unavoidable mechanical stress. In this regard, Li et al. utilized an electrostatic self-assembly strategy to incorporate an rGO interlayer sandwiched between poly (diallyldimethylammonium chloride) (PDDA)-modified fibre textile (PMFT) and PANI as the active material to prepare PANI@rGO/PMFT, and then developed a high-performance ASS supercapacitor by using PANI@rGO/PMFT as flexible textile electrodes and BCNF-reinforced polyacrylamide (BCNFs-PAM/H_2_SO_4_) as the gel electrolyte (Figure 10). The as-developed ASS supercapacitor exhibited high ionic conductivity of 125 mS/cm, high tensile strength of 330 kPa, and excellent elasticity (stretching up to ~1300%). Moreover, the ASS supercapacitor showed high areal capacitance (564 mF/cm^2^), remarkable rate capability, high energy/power densities, and superior mechanical performance without significant capacitance reduction after repeated bending [155].

As reviewed and comparatively discussed in the previous sections, various CNS-types used, their processing parameters, compositions (electrode-types), electrolytes, and their supercapacitive properties are summarized in Table 1.

## 5. Conclusions and Future Perspectives

Supercapacitors are expected to be promising candidates for numerous portable electronic devices and hybrid electric vehicles due to their long cycle life, safe and ultra-fast charging and discharging characteristics, and strong thermal operating temperature. However, expansion and contraction upon cycling are major drawbacks of polymer-based electrode materials. This causes deterioration of the electrode and structural damage, resulting in poor cycle stability due to volume changes during charging and discharging. The production of new composites from metal oxides or carbon-based materials can overcome these shortcomings. The carbon materials traditionally used in supercapacitors are mainly derived from fossil resources, so they face serious environmental problems and will suffer from the loss of fossil resources in the near future.

The remarkable recent developments in portable electronic devices have attracted researchers to the fabrication of low-cost, lightweight, eco-friendly, and adaptable energy storage devices with high supercapacitive performance. Sustainable energy storage devices are receiving significant attention in the provision of energy for a range of technical purposes. In these advancements, cellulose fibres as sustainable biomass have shown great potential due to their natural, abundance, lost-cost, renewability, flexibility, biodegradability, inherent biocompatibility, and eco-friendly aspects. These fibres are arranged in a 3D porous structure (i.e., fibrous-network) and have strong mechanical strength. There are large number of hydroxyl groups on their surfaces in addition to hydrogen bonding sites for strong interactions with other hydrophilic substrates. In all cellulose types, bacterial cellulose has more surface hydroxyl groups than plant-based cellulose and provides opportunities for functionalization. Although cellulose fiber is not itself conductive, it can be integrated with conductive materials to fabricate functional energy storage devices. However, the more efficient integration of cellulose with other substrate supports to create flexible supercapacitors is not easy task and need to be improved for better outcomes. There have been reported various methods for imparting conductivity to celluloses. Following this, nanocellulose-derived carbon is a more favorable functional material due to its low environmental effect, sustainability, and high specific surface area.

In this review, we demonstrate the efficacy and recent advances based on cellulose-derived nanostructures (e.g., CNCs, CNFs, and BCNFs) or their carbonized-forms in supercapacitor applications. However, these carbon materials typically contain mesopores and micropores, impeding the penetration and loading of conductive polymers. The fabrication of high-performance biomass-derived carbon/conductive polymer hybrids remains a major challenge. The challenges and opportunities in developing functional materials for sustainable supercapacitor applications were discussed and then the brief discussion on cellulose and cellulose-derived nanostructures (nanocelluloses) as sustainable biomass was presented. Cellulose-derived nanostructure-based functional electrode materials, including metallic particles, conductive polymers, conductive materials or their hybrids were reviewed and discussed, particularly regarding their supercapacitor applications. Nanocelluloses have outstanding characteristics and play a decisive role in developing multifunctional electrode materials for supercapacitors. Microsized electronic devices are considered more promising due to their microscale, flexibility, and light-weight energy conversion and storage capability. Among different microdevices, micro-supercapacitors are very effective energy storage devices owing to their ultra-high power density, rapid rechargeability, better rate capacity, and long cyclability [160]. Moreover, cellulose microfibers or nanostructures have great potential in developing flexible, wearable microsized and piezoelectric supercapacitors in the future [161,162].

## Figures and Tables

**Table 1:** Cellulose-derived nanostructure-based supercapacitors.

CNS-Type	Processing	Electrode-Type	Electrolyte	Specific Capacitance	Energy Density	Power Density	Capacitance Retention (%)	Ref.
CNFs	Electrospinning, regeneration, and carbonization (800 °C)	N-doped CBNFs/CA/SPI	6 M KOH	219.3 F/g at 0.2 A/g	5.6 W h/kg	16.8 kW/kg	98.9 after 50,000 cycles at 20 A/g	[142]
CNCs/CNFs	Carbonization (800 °C)	SSAD/CBNFs	6 M KOH	149 F/g at 0.25 A/g	20.64 W h/kg	0.25 kW/kg	500 cycles	[139]
CNFs	Carbonization (900 °C)	CBNFAs-17%	1 M H_2_SO_4_	440.29 F/g at 1.0 A/g	0.081 mW h/cm^2^		100 after 7000 cycles	[140]
BCNFs	Chemical mixing	CBNFs/PDA-Fe^2+^	1 M H_2_SO_4_	219.0 at 10.0 A/g	10.07 W h/kg	1.0 kW/kg	95 after 10,000 cycles	[141]
BCNFs	Submerging in NH_2_H_2_PO_4_, and then pyrolysis	N/P co-doped CBNFs	2 M H_2_SO_4_	204.9 F/g at 1.0 A/g	7.76 W h/kg	186.03 kW/kg	Very stable upto 4000 cycles	[129]
BCNFs		N/P/S co-doped CBNFs	2 M H_2_SO_4_	255.0 F/g at 1.0 A/g	8.48 W h/kg at 1.0 A/g	489.45 W/kg	slight change after 3500 cycles	[130]
Co_3_O_4_ NPs/CNFs	Carbonization at 200 °C for 20 min under H2/Ar ambience	Co_3_O_4_@CNFs	3 M KOH	∼214 F/g at 1.0 A/g	10 W h/kg		∼94 after 5000 cycles	[103]
ACNFs	Specimen was fabricated on the Al substrate via slip casting	N/A				13.1 W/kg	Cs = 1.85f^−0.494^ (r^2^ = 0.9984)	[102]
CNFs/zeolite	In-situ chemical process followed by pyrolysis	HZNPC & (1,3,5%) CNF-HZNPC coated GCE	1 M KOH	115 F/g & (130,147,101) at 1 A/g				[104]
TEMPO-CNFS/MnO_2_	Pyrolysis followed by hydrothermal process	TEMPO-CNFS/MnO_2_ and activated C		171.1 F/g at 0.5 A/g	8.6 W h/kg	619.2 W/kg	98.4% after 5000 cycles at 3 A/g	[109]
Ti_3_C_2_T_x_/CNFs	Vacuum filtration process	PC/PTFE/	PVA/KOH gel	143 mF cm^−2^ at 0.1 mA cm^−2^	2.4 µW h/cm^2^ at 17.5		50% after enhancing power density by 100 times	[137]
Ti_3_C_2_T_x_/CNFs	Delaminated Ti_3_C_2_T_x_ flakes modified by alkalization and annealing at 400 °C		3 M H_2_SO_4_	303.1 & 211.4 F/g at 1.0 & 10.0 mA/cm^2^			92.84% after 10,000 cycles	[138]
NPCNs/CNFs	Activating co-assisted carbonization process and vacuum filtration	NPCN-f and NPCN/MnO_2_-f	2 M KOH and 1 M Na_2_SO_4_	351, and 318 F/g for NPCN, and NPCN-f at 1 A/g	41.5 W h/kg	182.0 W/Kg	93% after 10,000 cycles	[131]
GO/CNCs	Non-liquid crystal spinning and chemical reduction	rGO/CNC-20	PVA/H_2_SO_4_ gel	208.2 F/cm^3^	5.1 W h/cm^3^	496.4 W/cm^3^	92.1% after 1000 cycles	[111]
HPC/NiCo_2_O_4_	Pump filtration process	HPC & HPC/NiCo_2_O_4_	6 M KOH	64.83 F/g & 32.78 F/g at 0.25 & 4 A/g	23.05 W h/kg	213 W/kg	96.8% after 1000 cycles at 10 A/g	[112]
J11/CNTs/BCNFs	One pot esterification process	CNTs/BCNFs & J11/CNTs/BCNFs	1 M H_2_SO_4_	461.8 F/cm^3^ at 10 A/g	41.9 W h/kg	1.0 KW/Kg	82.4% after 10,000 cycles at 10 A/g	[113]
Fe_3_O_4_/CNTs/CNCs	Hydrothermal reduction process	N doped MWCNT	2 M KOH	562 F/g at 0.5 A/g			94.6% after 5000 cycles	[114]
rGO/CBNFs	Low temperature thermal treatment	rGO350	6 M KOH	210 F/g at 10 A/g			97% after 100 cycles	[115]
rGO/SnO_2_/CNFs	Hydrothermal reduction process	CNFs/RGO/SnO_2_	1 M H_2_SO_4_	4.314 F/cm^2^ at 1 mA/cm^2^			60.47% after 2000 cycles at 10 mA/cm^2^	[116]
rGO/CNFs	Hummers process	GO/GO-CNF	PVA/H_3_PO_4_	120 mF/cm^2^	536 W h/kg	193 mW/cm^2^		[117]
Biomass carbon/rGO/CNFs	One-step self-assembly process	Bio-AC/rGO/CNF	1 M Na_2_SO_4_	4.8 F/cm^2^	365 mW h/cm^2^	18,000 mW/cm^2^	99% after 500 cycles	[118]
Graphene QD/CNFs	Electrolysis and liquid phase dispersion	CNF@GQD	1.5 M Li_2_SO_4_	118 mF/cm^2^	5961 W h/cm^2^	782 mW/cm^2^	>93% after 500 cycles	[119]
PANI/BCNFs	In situ polymerization	Acetylene/PTFE	1 M H_2_SO_4_	273 F/g at 2.0 A/g			94.3% after 500 cycles	[121]
PANI/CNFs	In situ polymerization	PANI/CNF/GNP	PVA/H_2_SO_4_	421.5 F/g at 1.0 A/g			78.3% after 1000 cycles	[122]
PPy/CBNFs/Ni(OH)_2_	Electrospinning, deacetylation and polymerization	N-CBNFs/CBNFs-Ni(OH)_2_	6 M KOH	1045 F/g	51 W h/Kg	117 kW/Kg	84% after 5000 cycles	[123]
PPy/TEMPO/BCNFs	In situ polymerization	PPy-TOBC	PVDF-EMIMBF_4_	153 F/g	21.22 W h/Kg		93% after 100 cycles	[124]
PEDOT/CNCs	Electrochemical polymerization	ITO/Pt/Ag/AgCl	1 M NaCl	117.02 F/g at 0.2 A/g	11.44 W h/Kg	99.85 W/Kg	86% after 1000 cycles	[128]
Graphene/PANI/BCNFs	Polymerization and filtration	PANI/GN/BC	1 M H_2_SO_4_	1.32 F/cm^2^	0.12 mW h/cm^2^		91.5% after 2000 cycles	[143]
Graphene/PANI/BCNFs	In situ polymerization	PANI/PG–BC4	PVA/H_2_SO_4_	1389 mF/cm^2^ at 2 mA/cm^2^	9.80 mW h/cm^3^	0.20 mW/cm^2^	89.8% after 5000 cycles	[144]
PPy/rGO/CNFs	Vacuum filtration and chemical reduction	PPy@rGO/CNFs	1 M H_2_SO_4_	625.6 F/g at 0.22 A/g	21.7 W h/Kg	0.11 kW/Kg	75.4% after 5000 cycles	[145]
TEMPO/rGO/PPy/CNFs	Vacuum filtration and chemical reduction	CNFs/RGO@PPy-2 FSCs	1 M H_2_SO_4_	647 F/g at 0.1 mA/cm^2^ at 0.1 mA/cm^2^	14.37 mW h/cm^2^	20 mW cm^−2^	92.6% after 10,000 cycles	[146]
TEMPO-NC/CNT/PS	Extraction and chemical oxidation of bleached wood pulp	TC-s-CNT-PS	1 M H_2_SO_4_	65 F/g			60% after 2000 cycles	[147]
PPy/rGO/TEMPO/BCNFs	Chemical mixing and sonication	PPy@TOBC/rGO	PVA/H_2_SO_4_	391 F/g at 0.5 A/g	4.1 mW h/cm^3^	429.3 mW/cm^3^	79% after 5000 cycles	[148]
PPy/CNT/BCNFs	Chemical mixing and In-situ polymerization	PPy@CNT/BC	PVA/H_2_SO_4_	228 F/g at 0.5 A/g	4.2 W h/kg	454.5 W/Kg	79% after 5000 cycles	[149]
PPy/Ni-Mn/BCNFs	In situ layered-by-layer deposition	BiVO_4_, BiVO_4_/Ni(OH)_x_, BiVO_4_/Mn(OH)_x_ or BiVO_4_/NiMn-LDH	0.5 M Na_2_SO_4_	653.1 F/g at 0.5 A/g	29.8 W h/kg	299 W/Kg		[150]
CNT/PVAB-CNFs	Chemical mixing and sonication	CNT-CNF/PVAB-2	H_3_PO_4_/PVA	117.1 F/g at 0.5 A/g			96.4% after 1000 cycles	[151]
MWCNT/PANI/CC/CNFs	In situ polymerization	CNFs/CNTs/PANI/CC	1 M H_2_SO_4_	318 F/g at 10 mA/s			72.09% after 1000 cycles	[152]
PAM/H_2_SO_4_/BCNFs	Electrostatic self-assembly approach	PANI/RGO/PMFT	BC/PAM	564 mF/cm^2^	50.1 μW h/cm^2^	20 mW/cm^2^	97.5% after 2000 cycles	[155]
NiS/BCNFs	Dissolution/gelation/carbonization	NiS/BC & BC	2 M KOH		21.5 W h/kg	700 W/kg	87.1% after 10,000 cycles	[156]
PPy/GO/BCNFs	Chemical mixing and pyrolyzation	N doped/RGO/BCNFs	6 M KOH/1 M H_2_SO_4_		0.1 mW h/cm^2^ in KOH & 0.1 mW h/cm^2^ in H_2_SO_4_	27.0 mW/cm^2^ in KOH & 37.5 mW/cm^2^ in H_2_SO_4_	99.6% after 10,000 cycles	[157]
PEDOT/CNFs	Hybrid formation through in situ polymerization	NFC@PEDOT	1 M H_2_SO_4_		1.1 mW h/cm^3^	900 mW/cm^2^	93% after 15,000 cycles	[158]
PANI/G/BCNFs	Hybrid formation with PANI and graphene	PANI/GO/BCNFs	1 M H_2_SO_4_	1.9 F/cm^2^ 0.25 mA/cm^2^	0.2 mW h/cm^2^	0.1 mW/cm^2^	53.6% after 5000 cycles	[159]

## Data Availability

The data presented in this study are available on request from the corresponding author.
